# Establishment and Validation of SSCLIP Scoring System to Estimate Survival in Hepatocellular Carcinoma Patients Who Received Curative Liver Resection

**DOI:** 10.1371/journal.pone.0129000

**Published:** 2015-06-09

**Authors:** Sha Huang, Gui-Qian Huang, Gui-Qi Zhu, Wen-Yue Liu, Jie You, Ke-Qing Shi, Xiao-Bo Wang, Han-Yang Che, Guo-Liang Chen, Jian-Feng Fang, Yi Zhou, Meng-Tao Zhou, Yong-Ping Chen, Martin Braddock, Ming-Hua Zheng

**Affiliations:** 1 Department of Infection and Liver Diseases, Liver Research Center, the First Affiliated Hospital of Wenzhou Medical University, Wenzhou 325000, China; 2 School of the First Clinical Medical Sciences, Wenzhou Medical University, Wenzhou 325000, China; 3 Renji School of Wenzhou Medical University, Wenzhou 325000, China; 4 Department of Endocrinology, the First Affiliated Hospital of Wenzhou Medical University, Wenzhou 325000, China; 5 Department of Oncological Surgery, the First Affiliated Hospital of Wenzhou Medical University, Wenzhou 325000, China; 6 Institute of Hepatology, Wenzhou Medical University, Wenzhou 325000, China; 7 Intensive Care Unit, Wenzhou Central Hospital, Wenzhou 325000, China; 8 Department of Hepatobiliary Surgery, the Second Affiliated Hospital of Wenzhou Medical University, Wenzhou 325000, China; 9 Department of General Surgery, Jinhua People’s Hospital, Jinhua 321000, China; 10 Department of Hepatobiliary Surgery, Shaoxing People’s Hospital, Shaoxing Hospital of Zhejiang University, Shaoxing 312000, China; 11 Department of Hepatobiliary Surgery, the First Affiliated Hospital of Wenzhou Medical University, Wenzhou 325000, China; 12 Global Medicines Development, AstraZeneca R&D, Alderley Park, Macclesfield, United Kingdom; Taipei Veterans General Hosptial, TAIWAN

## Abstract

**Background and Aims:**

There is no prognostic model that is reliable and practical for patients who have received curative liver resection (CLR) for hepatocellular carcinoma (HCC). This study aimed to establish and validate a Surgery-Specific Cancer of the Liver Italian Program (SSCLIP) scoring system for those patients.

**Methods:**

668 eligible patients who underwent CLR for HCC from five separate tertiary hospitals were selected. The SSCLIP was constructed from a training cohort by adding independent predictors that were identified by Cox proportional hazards regression analyses to the original Cancer of the Liver Italian Program (CLIP). The prognostic performance of the SSCLIP at 12 and 36-months was compared with data from existing models. The patient survival distributions at different risk levels of the SSCLIP were also assessed.

**Results:**

Four independent predictors were added to construct the SSCLIP, including age (HR = 1.075, 95%CI: 1.019–1.135, P = 0.009), albumin (HR = 0.804, 95%CI: 0.681–0.950, P = 0.011), prothrombin time activity (HR = 0.856, 95%CI: 0.751–0.975, P = 0.020) and microvascular invasion (HR = 19.852, 95%CI: 2.203–178.917, P = 0.008). In both training and validation cohorts, 12-month and 36-month prognostic performance of the SSCLIP were significantly better than those of the original CLIP, model of end-stage liver disease-based CLIP, Okuda and Child-Turcotte-Pugh score (all P < 0.05). The stratification of risk levels of the SSCLIP showed an enhanced ability to differentiate patients with different outcomes.

**Conclusions:**

A novel SSCLIP to predict survival of HCC patients who received CLR based on objective parameters may provide a refined, useful prognosis algorithm.

## Introduction

Hepatocellular carcinoma (HCC) is the third leading cause of death in patients with malignancies in the world [1, 2]. Curative liver resection (CLR) provides early HCC patients with a radical therapy although overall prognosis post surgery is still an issue of great concern. The Cancer of the Liver Italian Program (CLIP) scoring system is a widely accepted prognostic model for patients with HCC. To date, it remains controversial whether it is the best model for predicting the prognosis in patients who underwent CLR for HCC [3, 4].

Up until now, there are two principal modified CLIP scoring systems. These include the modified CLIP using protein induced by vitamin K absence or antagonist II (PIVKA-II) [5] and the model of end-stage liver disease (MELD)-based CLIP [6]. Unfortunately, neither of the scoring systems has been validated in other independent medical centers. Meanwhile, only the modified CLIP using PIVKA-II is aimed at patients who undergo CLR for HCC, and PIVKA-IIis not a commonly used factor that is available for all HCC patients. There is therefore a great need to develop a reliable prognostic model for HCC patients who received CLR with widely available variables.

The aim of this study was to construct and validate a modified CLIP scoring system specific to patients who underwent CLR for HCC in two large separated cohorts involving five independent tertiary medical centers so as to produce accurate prognostic information after surgery.

## Materials and Methods

### Study Design

In this study, we established and validated a modified CLIP scoring system named Surgery-Specific CLIP (SSCLIP) by recruiting patients who received CLR for HCC from five separated tertiary medical centers (training cohort: the First Affiliated Hospital of Wenzhou Medical University from January 2005 to June 2010; validation cohort: the Second Affiliated Hospital of Wenzhou Medical University; Jinhua People’s Hospital; Shaoxing People’s Hospital; Wenzhou Central Hospital; from September 2008 to January 2014).

The start date of the follow-up was the date of CLR. The follow-up periods were 46.3 ± 25.2 months in the training cohort and 24.7 ± 15.9 months in the validation cohort. Written informed consent was obtained from each patient included in the study and the research protocol of the study was approved by the Ethics Committee of the First Affiliated Hospital of Wenzhou Medical University, the Second Affiliated Hospital of Wenzhou Medical University, Jinhua People’s Hospital, Shaoxing People’s Hospital and Wenzhou Central Hospital.

### Inclusion and Exclusion Criteria

Patients who received CLR for suspected HCC were selected. The diagnosis of HCC was verified by post-operative interpretation of pathological analyses and patients who met the following criteria were excluded: 1) non-HCC diseases according to postoperative pathological diagnosis; 2) not the first primary cancer; 3) multiple primary cancer; 4) distant metastasis; 5) previous history of hepatic resection; 6) previous history of percutaneous ethanol injection, radiofrequency, transarterial chemoembolization or liver transplantation.

### Data Collection and Follow-up

Patient demographic information, HCC etiology, clinical and laboratory data within the week before CLR were extracted from medical records. Demographic information included age and gender. Clinical parameters included hepatic encephalopathy (HE), history of alcohol abuse, liver cirrhosis (LC) and ascites. HE was diagnosed according to West-Haven criteria [7]. Ascites were detected by physical examination and confirmed by ultrasonic test, computerized tomography (CT) or magnetic resonance imaging (MRI). LC was detected by ultrasound, CT or MRI, and confirmed by histological examination. Laboratory parameters included total bilirubin (TB), direct bilirubin (DB), albumin (ALB), alanine aminotransferase (ALT), aspartate aminotranferase (AST), alkaline phosphatase, γ-glutamyl transferase (γ-GT), blood glucose, serum creatinine (Cr), uric acid, serum sodium, prothrombin time (PT), prothrombin time activity (PTA), international normalized ratio (INR), white blood cell count, platelet count, and hepatitis B virus (HBV) serologic markers (Abbott, AXSYM). Hepatitis C virus (HCV) antibody was detected using ELISA (IEGAN, Freedom evolyzer/150). Tumor characteristics including the number of tumor nodules, the diameter of the largest nodule and portal vein thrombosis were observed during the surgery. Microvascular invasion (MVI) was observed in postoperative pathologic examination. Alpha-fetoprotein (AFP) level within the week before CLR was collected.

Patients were followed-up every 3 months after surgery. At follow-up, CT or MRI imaging was performed and the level of AFP was assessed. Information on death was obtained from the social security death index and medical records along with notifications from the family of the deceased were used to supplement the information to ensure complete capture of all decedents.

### Scoring Systems and Prognostic Models

The model of end-stage liver disease (MELD) score was calculated according to the Malinchoc formula: MELD = 9.57 × ln(creatinine [mg/dL]) + 3.78 × ln(bilirubin [mg/dL]) + 11.2 × ln(INR) + 6.43 × (aetiology: 0 if cholestatic or alcoholic, 1 otherwise) [8]. Okuda score, which included tumor size, ascite, TB, ALB, was assessed and divide into 3 stages according to the standard criteria [9]. CTP score, which included HE, ascite, TB, ALB and PT, was assessed and divide into 3 stages according to the standard criteria [10]. Original CLIP score, which included CTP stage, tumor morphology, AFP, and portal vein thrombosis, was assessed according to standard criteria [11]. MELD-based CLIP score, which replaced the CTP score with the MELD score in the original CLIP, was calculated according to Huo et al [6].

### Construction of the SSCLIP

In the training cohort, we performed univariate Cox proportional hazards regression analysis for determining the association of demographic, clinical, laboratory parameters and tumor characteristics with prognosis and survival time. Then, those covariables with univariate significance (P < 0.05) were included in a multivariate Cox proportional hazards regression to identify independent predictors for the prognosis of the patients with HCC. These independent predictors were added into the original CLIP to construct the SSCLIP in which receiver operating characteristics (ROC) analysis or recognized standard was used to identify possible predictive cutoff values for the predictors. Different sets of cutoff values and possible combinations were tested and optimal combination was chose in terms of outcome evaluation.

To assess 12-month and 36-month prognostic efficiency of the SSCLIP, the area under the receiver operating characteristic curve (AUROC) was calculated both in the training and validation cohorts. Comparison of the AUROC between the SSCLIP and other models was performed using the method of Hanley and McNeil [12]. The survival distributions of different score categories were assessed by the Kaplan-Meier method and compared using a log rank test. The standard indices of validity, such as sensitivity, specificity, positive predictive value (PPV), negative predictive value (NPV) were calculated according to the ROC results.

### Statistical Analysis

Continuous variables were expressed as mean ± standard deviation (SD) and categorical values were expressed by absolute and relative frequencies. Differences in continuous variables were analyzed using Mann-Whitney U test. The Chi-square test or the Fisher’s exact test was used for categorical data as appropriate. For all analyses, a P value of < 0.05 was considered statistically significant. Statistical analysis was performed using the IBM SPSS Statistics Version 19.0 (IBM Corp, Armonk, NY, USA) and MedCalc version 13.0 (MedCalc Software, Ostend, Belgium).

## Results

### Baseline Characteristics of Included Patients

**Fig 1:**
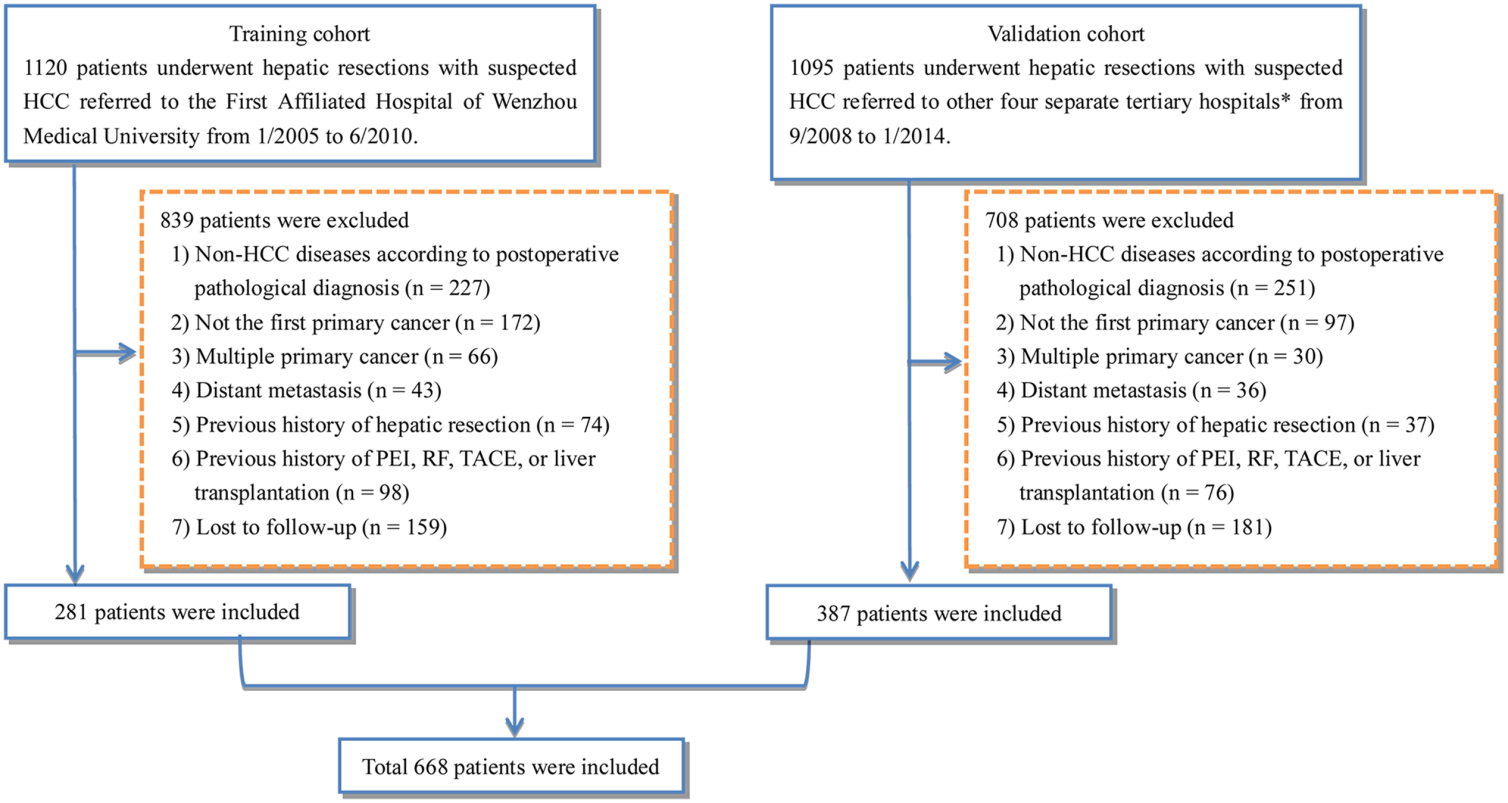
A flow diagram of study participants. *the Second Affiliated Hospital of Wenzhou Medical University, Jinhua People’s Hospital, Shaoxing People’s Hospital, Wenzhou Central Hospital; HCC, hepatocellular carcinoma; PEI, Percutaneous ethanol injection; RF, radiofrequency; TACE, Transarterial chemoembolization.

2215 patients received CLR for suspected HCC were enrolled in the study. After exclusion of those patients who did not meet the inclusion criteria (Fig 1), 668 patients (281 in the training cohort and 387 in the validation cohort) were finally included. Characteristics collected were presented in Table 1. Mean age of included patients was 55.8 ± 11.3 years in the training cohort and 57.0 ± 10.9 years in the validation cohort and the patients were predominantly men (86.1% in the training cohort and 84.5% in the validation cohort). HBV was the predominant etiology in both cohorts (48.0% and 71.0%), followed by superinfection of HBV and HCV (36.2% and 12.8%). Both cohorts did not include patients with HE. The majority of patients had a single tumor (87.2% and 89.0%), with tumor diameter of 50.1 ± 33.5 mm and 47.4 ± 31.2 mm in the training and validation cohorts respectively.

**Table 1:** Characteristics of Hepatocellular Carcinoma Patients Who Received Curative Liver Resection, Stratified by Cohort.

Variables	Training cohort (n = 281)	Validation cohort (n = 387)	P-value
**Demographic parameters**			
Age (years)	55.8 ± 11.3	57.0 ± 10.9	0.156
Male gender, n (%)	242 (86.1%)	327 (84.5%)	0.560
**Clinical parameters**			
Ascites, n (%)	23 (9.9%)	32 (9.6%)	0.013
Liver cirrhosis, n (%)	121 (50.8%)	147 (43.8%)	0.390
**Etiology**			
HBV, n (%)	131 (48.0%)	272 (71.0%)	< 0.001
HCV, n (%)	3 (1.1%)	3 (0.8%)	0.375
Alcohol, n (%)	21 (7.7%)	31 (8.1%)	0.259
HBV + HCV, n (%)	99 (36.2%)	49 (12.8%)	< 0.001
Other, n (%)	19 (7.0%)	28 (7.3%)	0.277
**Laboratory parameters**			
Total bilirubin (μmol/L)	15.6 ± 21.0	14.0 ± 19.1	0.028
Direct bilirubin (μmol/L)	6.1 ± 14.8	6.8 ± 20.4	0.882
Albumin (g/L)	40.6 ± 5.8	39.9 ± 5.7	0.098
ALT (IU/L)	58.2 ± 78.0	53.6 ± 78.0	0.092
AST (IU/L)	67.7 ± 92.5	58.0 ± 118.0	0.069
Alkaline phosphatase (IU/L)	106.8 ± 53.9	110.1 ± 91.0	0.735
γ-GT (IU/L)	87.4 ± 80.0	98.7 ± 153.4	0.334
Blood glucose (mmol/L)	6.4 ± 2.9	6.6 ± 2.9	0.047
Creatinine (μmol/L)	69.8 ± 32.1	69.6 ± 22.6	0.651
Uric acid (μmol/L)	294.6 ± 85.2	304.9 ± 91.9	0.199
Serum sodium (mmol/L)	142.6 ± 24.1	140.3 ± 2.8	< 0.001
PT (s)	14.3 ± 1.3	14.2 ± 1.3	0.159
PTA (%)	86.5 ± 13.0	87.8 ± 13.7	0.127
INR	1.5 ± 5.8	1.4 ± 4.9	0.056
White blood cell (10^9^/L)	6.4 ± 6.4	5.9 ± 5.5	0.096
Platelet (10^9^/L)	135.3 ± 64.6	140.6 ± 63.9	0.244
HBsAg positive (%)	229 (83.3%)	307 (79.9%)	0.280
HBsAb positive (%)	36 (17.1%)	68 (17.7%)	0.842
HBeAg positive (%)	56 (26.4%)	97 (25.3%)	0.757
HBeAb positive (%)	158 (74.9%)	300 (78.1%)	0.369
HBcAb positive (%)	204 (96.2%)	367 (95.6%)	0.703
**Tumor Characteristics**			
Alpha fetoprotein (ng/ml)	2388.9 ± 9330.8	1677.9 ± 6751.4	0.396
Number of nodules			
1	225 (87.2%)	331 (89.0%)	0.479
2	19 (7.3%)	30 (8.1%)	0.817
3	12 (4.7%)	9 (2.4%)	0.134
≥ 4	2 (0.8%)	2 (0.5%)	0.701
Size of the largest nodule (mm)	50.1 ± 33.5	47.4 ± 31.2	0.280
Portal vein thrombosis, n (%)	2 (0.7%)	10 (3.0%)	0.058
Microvascular invasion, n (%)	110 (39.1%)	88 (23.0%)	< 0.001
**MELD score**	8.0 ± 2.3	8.4 ± 3.6	0.041
**Okuda score**	0.5 ± 0.6	0.4 ± 0.6	0.883
**Okuda stage**			
I, n (%)	140 (61.4%)	225 (68.0%)	0.910
II, n (%)	87 (38.2%)	101 (30.5%)	0.664
III, n (%)	1 (0.4%)	5 (1.5%)	0.999
**CTP score**	5.7 ± 1.1	5.6 ± 1.2	0.984
**CTP stage**			
A, n (%)	183 (81.7%)	272 (82.4%)	0.177
B, n (%)	38 (17.0%)	51 (15.5%)	0.126
C, n (%)	3 (1.3%)	7 (2.1%)	0.263
**CLIP score**	1.1 ± 1.2	1.1 ± 1.2	0.999

**NOTE**. HBV, hepatitis B virus; HCV, hepatitis C virus; ALT, alanine aminotranferase; AST, aspartate aminotranferase; γ-GT, γ-glutamyl transferase; PT, prothrombin time; PTA, prothrombin time activity; INR, international normalized ratio; HBsAg, hepatitis B s antigen; HBsAb, hepatitis B s antibody; HBeAg, hepatitis B e antigen; HBeAb, hepatitis B e antibody; HBcAb, hepatitis B c antibody; MELD, model for end-stage liver disease; CTP, Child-Turcotte-Pugh; CLIP, Cancer of The Liver Italian Program.

Table 2 shows that patients who survived in the training cohort had a younger age (54.2 years vs 57.3 years, P = 0.041), a lower rate of MVI (28.5% vs 50.3%, P < 0.001), a higher ALB (41.6 g/L vs 39.7 g/L, P = 0.002), a tendency of higher PTA (87.4% vs 85.6% P = 0.289), a lower Okuda score (0.3 vs 0.5, P = 0.005), a lower CTP score (5.4 vs 5.7, P = 0.013), and a lower CLIP score (0.8 vs 1.3, P = 0.019). These conditions were similar to the validation cohort, except for PTA, which was significantly higher in survivors in the validation cohort (88.8% vs 83.5%, P = 0.002), and age (56.7 years vs 57.9 years, P = 0.431).

**Table 2:** Characteristics of Hepatocellular Carcinoma Patients Who Received Curative Liver Resection, Stratified by Mortality.

Variables	Training cohort (n = 281)*			Validation cohort (n = 387)^#^		
	Survival (n = 134)	Death (n = 147)	P-value	Survival (n = 317)	Death (n = 70)	P-value
**Demographic parameters**						
Age (years)	54.2 ± 11.4	57.3 ± 11.2	0.041	56.7 ± 11.1	57.9 ± 10.3	0.431
Male gender, n (%)	114 (85.1%)	128 (87.1%)	0.628	267 (84.2%)	60 (85.7%)	0.756
**Clinical parameters**						
Ascites, n (%)	2 (1.8%)	7 (6.1%)	0.100	25 (9.0%)	7 (13.0%)	0.366
Liver cirrhosis, n (%)	33 (28.9%)	57 (47.1%)	0.004	123 (44.1%)	24 (42.1%)	0.784
**Etiology**						
HBV, n (%)	62 (47.7%)	69 (48.2%)	0.698	242 (77.1%)	30 (43.5%)	< 0.001
HCV, n (%)	3 (2.3%)	0 (0%)	0.999	3 (1.0%)	0 (0%)	0.999
Alcohol, n (%)	7 (5.4%)	14 (9.8%)	0.236	23 (7.3%)	8 (11.6%)	0.023
HBV + HCV, n (%)	50 (38.5%)	49 (34.3%)	0.633	25 (7.9%)	24 (34.8%)	< 0.001
Other, n (%)	8 (6.1%)	11 (7.7%)	0.670	21 (6.7%)	7 (10.1%)	0.038
**Laboratory parameters**						
Total bilirubin (μmol/L)	12.5 ± 6.9	18.5 ± 28.0	0.008	13.1 ± 12.3	18.0 ± 36.4	0.677
Direct bilirubin (μmol/L)	4.0 ± 2.4	7.9 ± 20.0	0.001	4.1 ± 2.2	9.7 ± 29.2	0.139
Albumin (g/L)	41.6 ± 5.8	39.7 ± 5.6	0.002	40.2 ± 5.5	38.2 ± 6.4	0.011
ALT (IU/L)	54.3 ± 70.1	61.8 ± 84.6	0.076	49.6 ± 67.3	71.5 ± 113.5	0.011
AST (IU/L)	51.7 ± 66.8	82.3 ± 109.4	< 0.001	53.6 ± 118.8	82.3 ± 111.0	0.003
Alkaline phosphatase (IU/L)	95.3 ± 31.0	118.3 ± 68.1	0.101	106.9 ± 94.4	128.8 ± 65.0	< 0.001
γ-GT (IU/L)	72.6 ± 74.6	102.2 ± 83.0	0.003	98.7 ± 163.3	98.9 ± 72.6	0.007
Blood glucose (mmol/L)	6.6 ± 2.9	6.3 ± 2.9	0.164	6.6 ± 2.8	6.7 ± 3.1	0.366
Creatinine (μmol/L)	67.9 ± 23.1	71.5 ± 38.5	0.239	70.5 ± 22.8	65.2 ± 21.7	0.011
Uric acid (μmol/L)	291.9 ± 81.3	297.1 ± 88.8	0.757	307.5 ± 89.3	293.3 ± 103.0	0.149
Serum sodium (mmol/L)	141.1 ± 2.8	143.9 ± 33.1	0.602	140.3 ± 2.8	140.5 ± 2.8	0.330
PT (s)	14.2 ± 1.2	14.4 ± 1.3	0.262	14.1 ± 1.3	14.7 ± 1.4	0.001
PTA (%)	87.4 ± 12.8	85.6 ± 13.1	0.289	88.8 ± 13.6	83.5 ± 13.7	0.002
INR	1.1 ± 0.2	1.8 ± 8.0	0.184	1.1 ± 0.1	2.5 ± 11.6	< 0.001
White blood cell (10^9^/L)	5.6 ± 2.0	7.1 ± 8.6	0.188	5.5 ± 2.3	7.9 ± 11.9	0.110
Platelet (10^9^/L)	131.1 ± 59.2	139.1 ± 69.1	0.552	139.9 ± 62.8	143.5 ± 69.5	0.878
HBsAg positive (%)	111 (84.7%)	118 (81.9%)	0.536	255 (81.0%)	52 (75.4%)	0.294
HBsAb positive (%)	15 (13.9%)	21 (20.4%)	0.210	51 (16.2%)	17 (24.6%)	0.096
HBeAg positive (%)	24 (22.2%)	32 (30.8%)	0.158	74 (23.5%)	23 (33.3%)	0.088
HBeAb positive (%)	83 (76.9%)	75 (72.8%)	0.499	245 (77.8%)	55 (79.7%)	0.725
HBcAb positive (%)	105 (97.2%)	99 (95.2%)	0.678	299 (94.9%)	68 (98.6%)	0.315
**Tumor Characteristics**						
Alpha fetoprotein (ng/ml)	1764.3 ± 8995.1	2940.0 ± 9616.7	0.846	1248.7 ± 5038.0	3612.1 ± 11565.2	0.136
Number of nodules						
1	112 (90.3%)	113 (84.3%)	0.461	279 (90.3%)	52 (82.5%)	0.149
2	9 (7.3%)	10 (7.5%)	0.840	23 (7.4%)	7 (11.1%)	0.284
3	3 (2.4%)	9 (6.7%)	0.109	5 (1.6%)	4 (6.4%)	0.034
≥ 4	0 (0%)	2 (1.5%)	0.999	2 (0.7%)	0 (0%)	0.999
Size of the largest nodule (mm)	47.1 ± 30.0	53.1 ± 35.7	0.238	45.3 ± 29.3	57.7 ± 37.7	0.010
Portal vein thrombosis, n (%)	0 (0%)	1 (0.9%)	1.000	7 (2.5%)	3 (5.6%)	0.447
Microvascular invasion, n (%)	37 (28.5%)	73 (50.3%)	< 0.001	48 (15.3%)	40 (58.0%)	< 0.001
**MELD score**	7.9 ± 1.6	9.0 ± 5.2	0.121	7.9 ± 2.0	9.4 ± 6.7	0.006
**Okuda score**	0.3 ± 0.5	0.5 ± 0.6	0.005	0.4 ± 0.6	0.6 ± 0.7	0.013
**Okuda stage**						
I, n (%)	83 (76.1%)	67 (58.8%)	0.006	196 (70.8%)	29 (53.7%)	0.052
II, n (%)	26 (23.9%)	47 (41.2%)	0.006	77 (27.8%)	24 (44.4%)	0.015
III, n (%)	0 (0%)	0 (0%)	-	4 (1.4%)	1 (1.9%)	0.644
**CTP score**	5.4 ± 0.8	5.7 ± 1.1	0.013	5.6 ± 1.2	6.1 ± 1.5	0.002
**CTP stage**						
A, n (%)	100 (93.5%)	92 (82.9%)	0.120	234 (84.5%)	38 (71.7%)	0.087
B, n (%)	7 (6.5%)	17 (15.3%)	0.040	38 (13.7%)	13 (24.5%)	0.042
C, n (%)	0 (0%)	2 (1.8%)	0.999	5 (1.8%)	2 (3.8%)	0.292
**CLIP score**	0.8 ± 1.0	1.3 ± 1.3	0.019	1.0 ± 1.2	1.5 ± 1.5	0.011

**NOTE**.

*The follow-up period of training cohort was 46.3 ± 25.2 months;

^#^The follow-up period of validation cohort was 24.7 ± 15.9 months; HBV, hepatitis B virus; HCV, hepatitis C virus; ALT, alanine aminotranferase; AST, aspartate aminotranferase; γ-GT, γ-glutamyl transferase; PT, prothrombin time; PTA, prothrombin time activity; INR, international normalized ratio; HBsAg, hepatitis B s antigen; HBsAb, hepatitis B s antibody; HBeAg, hepatitis B e antigen; HBeAb, hepatitis B e antibody; HBcAb, hepatitis B c antibody; MELD, model for end-stage liver disease; CTP, Child-Turcotte-Pugh; CLIP, Cancer of The Liver Italian Program.

### Construction of the SSCLIP

To identify independent predictors of mortality, the univariate and multivariate Cox proportional hazard analyses were performed in the training cohort (Table 3). In univariate Cox proportional hazards analysis, we found that age, LC, TB, DB, ALB, AST, alkaline phosphatase, γ-GT, PT, PTA, white blood cell count, number of tumor nodules, portal vein thrombosis and MVI were significantly associated with mortality (all P < 0.05). The above variables with univariate significance were then entered into the multivariate Cox proportional hazards regression analyses to select independent predictors. Age (HR = 1.075, 95%CI: 1.019–1.135, P = 0.009), ALB (HR = 0.804, 95%CI: 0.681–0.950, P = 0.011), PTA (HR = 0.856, 95%CI: 0.751–0.975, P = 0.020) and MVI (HR = 19.852, 95%CI: 2.203–178.917, P = 0.008) were found to be independent predictors.

**Table 3:** Univariate and Multivariate Cox Proportional Hazards Regression Analyses of the Associations between Mortality and Variables in the Training Cohort.

Variables	Univariate analysis				Multivariate analysis			
	B	HR	95% CI	P-value	B	HR	95% CI	P-value
**Demographic parameters**								
Age (years)	0.014	1.015	1.000–1.029	0.045	0.073	1.075	1.019–1.135	0.009
Gender	-0.044	0.957	0.591–1.550	0.859				
**Clinical parameters**								
Ascites	0.333	1.395	0.937–2.078	0.101				
Liver cirrhosis	0.537	1.711	1.195–2.449	0.003				
**Laboratory parameters**								
Total bilirubin (μmol/L)	0.005	1.005	1.001–1.010	0.027				
Direct bilirubin (μmol/L)	0.007	1.007	1.001–1.013	0.030				
Albumin (g/L)	-0.060	0.942	0.916–0.969	0.001	-0.218	0.804	0.681–0.950	0.011
ALT (IU/L)	0.001	1.001	1.000–1.003	0.126				
AST (IU/L)	0.003	1.003	1.001–1.005	0.006				
Alkaline phosphatase (IU/L)	0.009	1.009	1.005–1.013	0.001				
γ-GT (IU/L)	0.003	1.003	1.001–1.005	0.017				
Blood glucose (mmol/L)	-0.010	0.990	0.929–1.054	0.744				
Creatinine (μmol/L)	0.003	1.003	0.997–1.008	0.330				
Uric acid (μmol/L)	0.001	1.001	0.999–1.002	0.620				
Serum sodium (mmol/L)	0.003	1.003	0.998–1.008	0.233				
PT (s)	0.133	1.142	1.011–1.290	0.033				
PTA (%)	-0.014	0.986	0.974–0.999	0.030	-0.155	0.856	0.751–0.975	0.020
INR	0.007	1.007	0.987–1.028	0.475				
White blood cell (10^9^/L)	0.025	1.025	1.010–1.041	0.001				
Platelet (10^9^/L)	0.001	1.001	0.999–1.004	0.309				
HBsAg positive	-0.079	0.924	0.614–1.391	0.706				
HBsAb positive	0.320	1.377	0.853–2.226	0.191				
HBeAg positive	0.399	1.490	0.981–2.264	0.062				
HBeAb positive	-0.090	0.914	0.591–1.412	0.685				
HBcAb positive	-0.333	0.717	0.292–1.761	0.468				
**Tumor Characteristics**								
Alpha fetoprotein (ng/ml)	0.000	1.000	1.000–1.000	0.112				
Number of nodules	0.367	1.444	1.093–1.906	0.010				
Size of the largest nodule (mm)	0.004	1.004	0.999–1.009	0.085				
Portal vein thrombosis	3.602	36.688	4.417–304.742	0.001				
Microvascular invasion	0.831	2.296	1.651–3.192	0.001	2.988	19.852	2.203–178.917	0.008

**NOTE**. B, intercept; HR, hazard ratio; CI, confidence interval; ALT, alanine aminotranferase; AST, aspartate aminotranferase; γ-GT, γ-glutamyl transferase; PT, prothrombin time; PTA, prothrombin time activity; INR, international normalized ratio; HBsAg, hepatitis B s antigen; HBsAb, hepatitis B s antibody; HBeAg, hepatitis B e antigen; HBeAb, hepatitis B e antibody; HBcAb, hepatitis B c antibody.

Finally, those four independent predictors were added into the original CLIP to construct the SSCLIP. After testing different combinations of possible cut-off values, the cut-off values of age and ALB identified by ROC analysis were included, while the cut-off value of PTA was identified according to Asian-Pacific Association for The Study of Liver Guideline. As a result, patients older than 42 years, with an ALB level ≤ 41.6 g/L, a PTA < 40%, or MVI were given 1 additional score. The comparison between the original CLIP and the SSCLIP is shown in Table 4.

**Table 4:** The Original CLIP and the SSCLIP Scoring Systems.

Parameters	Score for the original CLIP	Score for the SSCLIP
**CTP class**		
A	0	0
B	1	1
C	2	2
**Tumor morphology**		
Uninodular and ≤ 50% liver span	0	0
Multinodular and ≤ 50% liver span	1	1
Massive or > 50% liver span	2	2
**AFP (ng/mL)**		
< 400	0	0
≥ 400	1	1
**Portal vein thrombosis**		
No	0	0
Yes	1	1
**Microvascular invasion**		
No	-	0
Yes	-	1
**Age (years)**		
≤ 42	-	0
> 42	-	1
**ALB (g/L)**		
> 41.6	-	0
≤ 41.6	-	1
**PTA (%)**		
≥ 40	-	0
< 40	-	1

**NOTE.** The score range is 0–6 for the original CLIP scoring system, 0–10 for the SSCLIP scoring system.

### Comparison between the SSCLIP and Other Models

**Fig 2:**
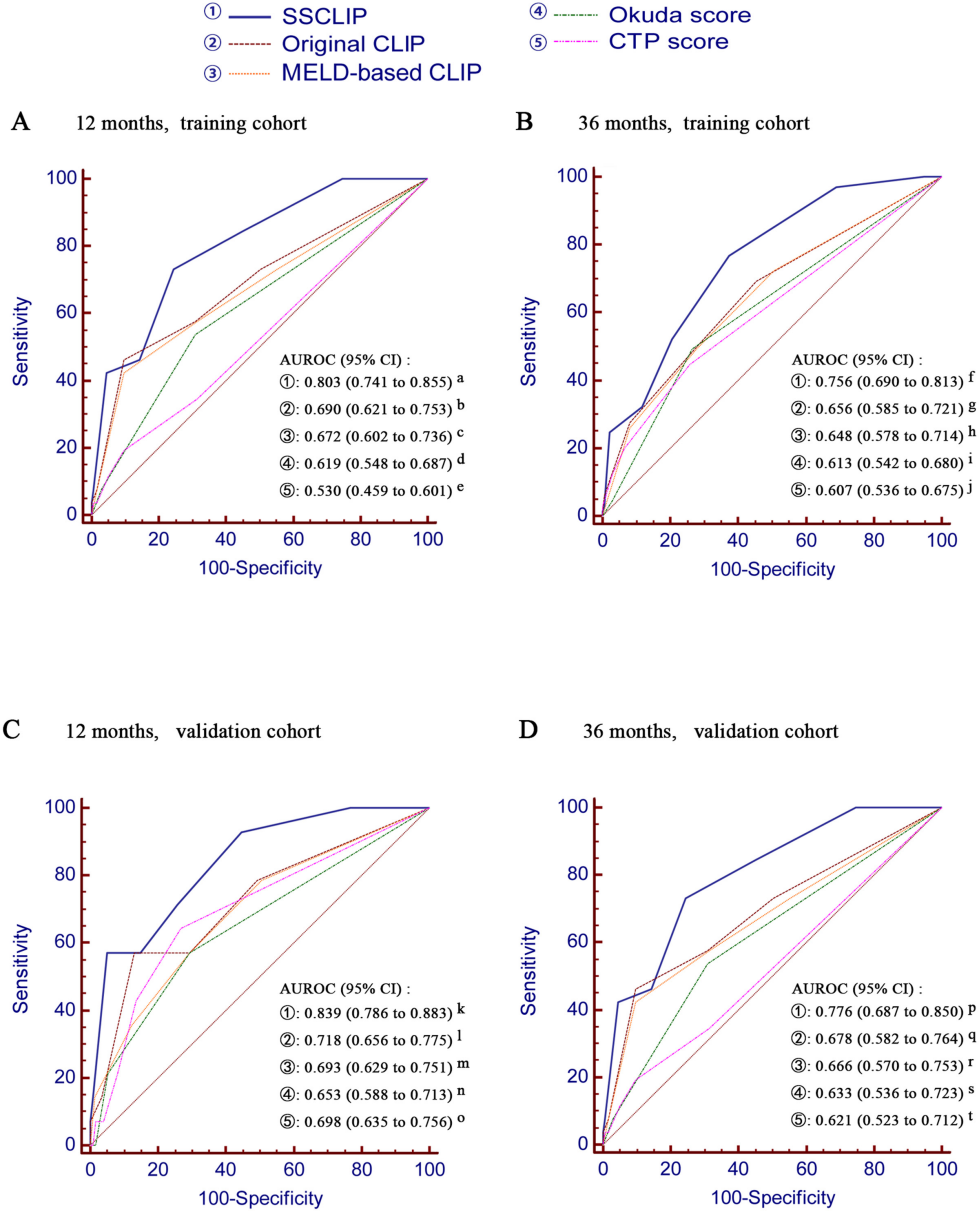
Comparison of the area under the receiver operating characteristic curve for prediction of mortality among different scoring systems in training cohort (panel A, at 12-month; panel B, at 36-month), and in validation cohort (panel C, at 12-month; panel D, at 36-month). P values: (1) Panel A: a vs b = 0.0004; a vs c = 0.0004; a vs d < 0.0001; a vs e < 0.0001; (2) Panel B: f vs g < 0.0001; f vs h < 0.0001; f vs i < 0.0001; f vs j = 0.0031; (3) Panel C: k vs l = 0.0020; k vs m = 0.0008; k vs n = 0.0003; k vs o = 0.0418; (4) Panel D: p vs q = 0.0004; p vs r = 0.0008; p vs s = 0.0001; p vs t = 0.0057. AUROC, area under the receiver operating characteristic curve; CI, confidence interval; CLIP, Cancer of The Liver Italian Program; MELD, model for end-stage liver disease; CTP, Child-Turcotte-Pugh.

The ability of the SSCLIP to predict 12-month and 36-month mortality risks was assessed in the training cohort by performing ROC analyses (Fig 2A and 2B). The AUROC was 0.803 for 12-month and 0.756 for 36-month, which were both significantly higher than the AUROCs of original CLIP (0.690 and 0.656), MELD-based CLIP (0.672 and 0.648), Okuda score (0.619 and 0.613) and CTP score (0.530 and 0.607) (all P < 0.05). When using a best cut-off value of 3 for 12-month prediction and 2 for 36-month prediction, the sensitivities were 73.08% and 76.92% respectively, the specificities were 75.43% and 62.50% respectively, the PPVs were 30.6% and 49.5% respectively and the NPVs were 95.0% and 85.0% respectively (Table 5).

**Table 5:** Diagnostic Accuracy of the SSCLIP Scoring System in the Training Cohort at Cut-off Points and at Different Time Periods.

	Cut-off point	All patients	Survival	Death	Se % (95% CI)	Sp % (95% CI)	PPV % (95% CI)	NPV % (95% CI)
12-month		n = 201	n = 175	n = 26				
	≤3	139	132	7				
	>3	62	43	19	73.08 (52.2–88.4)	75.43 (68.4–81.6)	30.6 (19.5–43.8)	95.0 (89.9–98.0)
36-month		n = 201	n = 136	n = 65				
	≤2	100	85	15				
	>2	101	51	50	76.92 (64.8–86.5)	62.50 (53.8–70.6)	49.5 (39.4–59.6)	85.0 (76.5–91.4)

**NOTE.** Se, sensitivity; Sp, specificity; PPV, positive predictive value; NPV, negative predictive value; CI, confidence interval.

As shown in Fig 2C and 2D, the robustness of the SSCLIP to predict mortality risks was tested externally in the validation cohort. The AUROC was 0.839 for 12-month and 0.776 for 36-month, both of which were still significantly better than the AUROCs of original CLIP (0.718 and 0.678), MELD-based CLIP (0.693 and 0.666), Okuda score (0.653 and 0.633), and CTP score (0.698 and 0.621) (all P < 0.05).

### Prediction of Survival in Different Risk Levels of the SSCLIP

**Fig 3:**
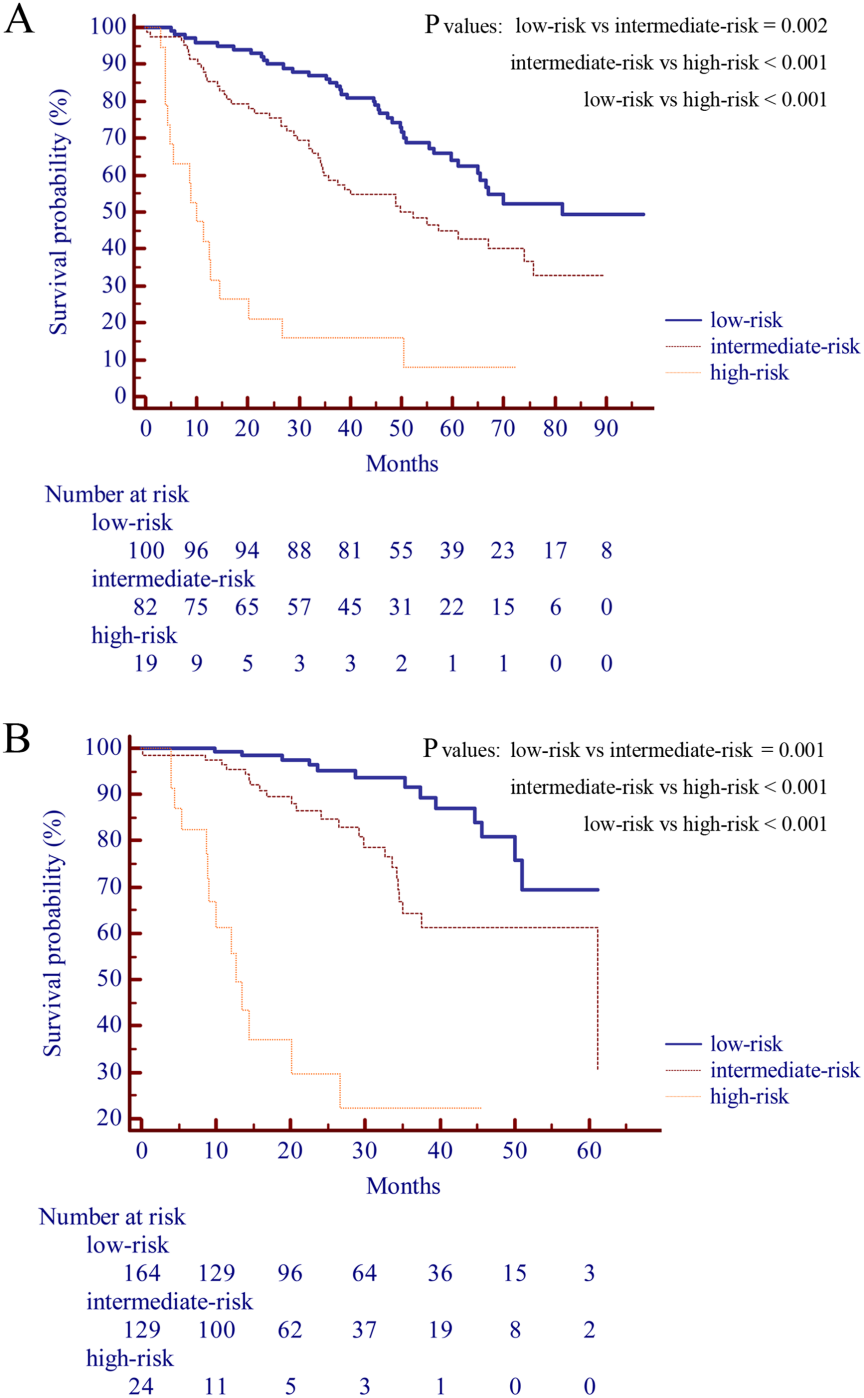
Survival distributions of different risk levels of the SSCLIP scoring system in the training cohort (panel A) and validation cohort (panel B). Low-risk, intermediate-risk and high-risk represent the SSCLIP score 0~2, 3~5 and ≥ 6 respectively.

We divided the score of the SSCLIP into three categories, which were low-risk, intermediate-risk and high-risk, standing for SSCLIP score 0~2, 3~5 and ≥ 6 respectively. The survival analysis was performed and survival distributions were compared between patients with different risk levels in the SSCLIP. In the training cohort (Fig 3A), patients in the low-risk level had more favorable survival after surgery than patients in the intermediate-risk level (χ^2^ = 9.221, P = 0.002), while those in high-risk level had poorer outcomes than intermediate-risk patients (χ^2^ = 26.665, P < 0.001), as well as low-risk patients (χ^2^ = 62.370, P < 0.001). This condition was confirmed in the validation cohort (Fig 3B), where low-risk patients had better outcomes than the intermediate-risk group (χ^2^ = 10.166, P = 0.001) and high-risk patients had poorer outcomes than the intermediate-risk patients (χ^2^ = 33.980, P < 0.001) and the low-risk patients (χ^2^ = 96.050, P < 0.001).

## Discussion

In this study, we established and validated a SSCLIP scoring system to predict both 12-month and 36-month prognosis of patients who underwent CLR for HCC. To our knowledge, this is the first surgery-specific modified CLIP scoring system that contained variables available in almost each HCC patients. After adding variables that were independent predictors to the original CLIP, the SSCLIP performed significantly better than the original CLIP, the MELD-based CLIP, the Okuda score and the CTP score, with an improvement of 12.6% to 14.4% over the original CLIP, and an improvement of 14.2% to 34.0% over the other predictive models. When divided into three risk levels, the SSCLIP showed a great ability to differentiate survival difference between different risk levels.

As previously known, in the original CLIP scoring system, factors of liver function were united as one item (CTP classification) which only counted for 2 scores at most, while there were three tumor characteristics which counted for 4 scores at the most included in the scoring system. This suggested that the CLIP scoring system mainly focused on tumor characteristics and might attach little weight on underlying liver function or other factors, when the prognosis of HCC might rely on both tumor characteristics and also other contributory factors. In the SSCLIP, we identified 4 more predictors which might provide more information about the patients’ status. These predictors, include 1 pathologic parameter, 1 clinical parameter and 2 laboratory parameters, were shown to be highly predictive not only in this study but also in previous studies and clinical practice. Previous studies had identified MVI as an independent risk factor that could affect mortality in patients who received CLR for HCC [13–16]. MVI had also proved to be a better predictor of tumor recurrence and overall survival following surgical resection for HCC compared with the Milan criteria which was discriminatory for selecting patients with good outcomes in liver transplantation and surgical resection for HCC [17]. In this study, MVI was again found as an independent risk factor and the patient survived in either of the cohorts had a significantly lower rate of MVI, which might indicate that it played an important role in the prognosis of patients who received CLR for HCC. Another factor identified in this study, age, was also an important factor. According to clinical experience, a younger age was usually associated with better liver function and other physical status and might be related to better recovery after surgery. This general observation supported performing the statistical analysis of age in this study. It was also shown by previous studies that a younger age was associated with a better survival rate after 1 year [18], and long-term survival rate in elderly patients undergoing hepatectomy for HCC were significantly lower than those in younger patients [15, 19–21]. In addition, we also found that the ALB level to be an independent predictor, which agreed with previous studies [22–26]. This was consistent with the fact that a declined ALB level could reflect liver dysfunction as ALB was synthesized in the liver. PTA level was another laboratory parameter we found to be an independent predictor. Previous studies had shown that the PTA level had an independent predictive value for the development and survival of HCC [27–29], and it is also widely accepted that the PTA level could reflect the levels of blood coagulation factors synthesized in the liver, that is, a lower PTA level might represent a poorer liver function.

In addition to identifying predictors to construct the SSCLIP, we also divided the score of the SSCLIP into three categories to represent different risk levels. As shown in Fig 3, survival rates were significantly higher in low-risk patients than in the other two levels whereas high-risk patients showed the lowest survival rates. This stratification of scores would be particularly useful in clinical practice because it may differentiate patients with a different prognosis and may guide the physician in choosing the most appropriate treatment or health care regime after surgery.

To date, most of the prognostic models of HCC have not been validated, partly because it is generally harder for a prognostic model to perform well in external cohorts than in the cohort from which it was derived. However, to ensure that the model could be applied in a wide range of patients, it was important to check the stability and reliability of the scoring system’s prognostic ability in different populations by performing external validation. In this study, we performed validation of the SSCLIP and generated data demonstrating the model’s good prognostic ability in an external cohort consisting of four independent medical centers. This finding strongly suggests that the SSCLIP is capable of being applied to a wide range of patients who underwent CLR for HCC.

There are, however, some limitations of this study. First, apart from the factors involved in this study, there might be other factors that were associated with the prognosis of HCC patients who underwent CLR. We regret that we were not able to involve all the potential factors in this retrospective study, especially some unusual factors that require special detection techniques. Nevertheless, since we intended to propose a practical and objective model, it was better to construct the SSCLIP with factors which could be easily assessed and would not be affected by subjective judgments. Secondly, the SSCLIP was derived from a population with included a majority of HBV-related HCC patients (including superinfection of HBV and HCV). Further studies are required to validate this model in populations where HCV, alcohol, non-alcoholic fatty liver disease, or other etiologies predominate. Thirdly, we also acknowledge that prospective studies with longer-term follow-up are needed to further extend the assessment of the SSCLIP’s performance.

In summary, we have established the first scoring system that was specific to patients who underwent CLR based on the CLIP with widely available variables. The SSCLIP scoring system may prove to be an ideal model useful in both epidemiologic research and in clinical practice for patient counseling and prognosis evaluation.
